# How we are misinterpreting physical activity intention – behavior relations and what to do about it

**DOI:** 10.1186/s12966-019-0829-y

**Published:** 2019-08-22

**Authors:** Amanda L. Rebar, Ryan E. Rhodes, Benjamin Gardner

**Affiliations:** 1 School of Health, Medical, and Applied Sciences, Central Queensland University, Building 18, Room 1.33, Bruce Highway, Rockhampton, Queensland 4703 Australia; 2 School of Exercise Science, Physical and Health Education, University of Victoria, Room 189, McKinnon Building, Victoria, BC V8W 3H5 Canada; 3 Department of Psychology, Institute of Psychiatry, Psychology, and Neuroscience, King’s College London, AH 2.11, Addison House, Guy’s Campus, London, SE1 1UL UK

**Keywords:** Habit, Assumption testing, Motivation, Simulation, Exercise, Goals

## Abstract

**Background:**

Studies of the physical activity intention-behavior gap, and factors that may moderate the gap (e.g., habit, perceived behavioral control), can inform physical activity promotion efforts. Yet, these studies typically apply linear modeling procedures, and so conclusions rely on linearity and homoscedasticity assumptions, which may not hold.

**Methods:**

We modelled and plotted physical activity intention-behavior associations and the moderation effects of habit using simulated data based on (a) normal distributions with no shared variance, (b) correlated parameters with normal distribution, and (c) realistically correlated and non-normally distributed parameters.

**Results:**

In the uncorrelated and correlated normal distribution datasets, no violations were unmet, and the moderation effects applied across the entire data range. However, because in the realistic dataset, few people who engaged in physical activity behavior had low intention scores, the intention-behavior association was non-linear, resulting in inflated linear moderation estimations of habit. This finding was replicated when tested with intention-behavior moderation of perceived behavioral control.

**Conclusions:**

Comparisons of the three scenarios illustrated how an identical correlation coefficient may mask different types of intention-behavior association and moderation effects. These findings highlight the risk of misinterpreting tests of the intention-behavior gap and its moderators for physical activity due to unfounded statistical assumptions. The previously well-documented moderating effects of habit, whereby the impact of intention on behavior weakens as habit strength increases, may be based on statistical byproducts of unmet model assumptions.

Physical inactivity is the fourth leading risk factor for global mortality – leading to an estimated 3.2 million preventable early deaths [1]. Despite ongoing global efforts to promote physical activity [2], most of the population in developed countries remains either entirely inactive or insufficiently active to optimally benefit from the physical and mental health benefits of regular activity (e.g., [3, 4]). There is a pressing need to understand how to effectively promote physical activity. Most individual-level interventions aim to promote physical activity through enhancing *intentions* – representations of self-instructions regarding either the direction of a goal (i.e., a goal to quit smoking), the intensity of commitment to act or not act (i.e., I strongly commit to my goal of quitting smoking), or a combination of both [5, 6].

Intention (in some form) is present in commonly applied theories to health behavior, including the theory of planned behavior [7], transtheoretical model [8], protection motivation theory [9], health action process approach [10], and social cognitive theory [11]. Typically, physical activity intentions are measured using continuous scales of the quantity of planned activity (i.e., *decisional intention*; ‘I intend to engage in __ minutes of physical activity next week’) or the degree of commitment a person has to enact their intention (i.e., *intention strength*; ‘To what degree do you intend to engage in physical activity next week?’: ‘Very little – Very much’) [5].

Intentions predict a substantial amount of variability of future physical activity duration and frequency [12–15]. In a meta-analysis of prospective correlational studies, McEachan et al. estimated that intentions explain 33% of variability in future physical activity behavior [14]. Experimental evidence also points to the importance of intention in predicting change in physical activity behavior, although to a smaller degree than seen in correlational studies [16, 17]. In their meta-analysis, Rhodes and Dickau found that experimental manipulations of activity intentions (*d* = 0.45) led only to small changes in physical activity (*d* = 0.15) [16]. The consistent intention-behavior link found in physical activity research suggests there is value in promoting intentions, but also illustrates that much variability in physical activity is not explained by intention. This disconnect has been coined the *intention-behavior gap* [15, 18]. Understanding behavior requires understanding not only intentions, but also factors that affect the likelihood of acting on intentions.

The process of translating intentions into behavior has been described as *action control* [15, 19]. To understand what factors contribute to action control of health behaviors, researchers have investigated person, context, and state variables that may moderate the magnitude of intention-behavior relationships. Such variables have included characteristics of intention itself (e.g., intention stability) [20], and more conceptually distinct factors, such as moral norms [21], habit [22, 23], self-efficacy [24], planning [25], and executive functioning [26], amongst others [19].

Investigations into moderators of the impact of intention on future behavior have both theoretical and applied merit, but they typically use linear modeling. Linear models make assumptions about how data are structured that have not been adequately tested for physical activity intention-behavior relationships. Linear modeling can be misleading if there are digressions from these assumptions [27–29]. For example, a simple but essential assumption of linear regression is *homoscedasticity* – that there is constant variance across the range of residuals for each predictor variable, such that the variability left unexplained by the model is the same for people with low and high scores [27, 29]. Another major assumption is that relationships between the outcome variable and the predictor variables within the regression model are linear, in that direct associations hold true for people with high and low values of both variables. Violations of assumptions of homoscedasticity or linearity result in misleading estimates of the magnitude and statistical significance of regression coefficients [28]. Even if the true effect does withstand the assumption violations, there is a high likelihood the findings of such models may only hold for a specific sub-group of the sample, so not be fully representative of the target population [27, 29]. In the real world, the distributions of physical activity intention and behavior data tend to be asymmetrical, resulting in non-normal model error distributions and non-linear relationships [19], so not meeting assumptions of linear modeling. Such methodological concerns are not trivial; seemingly small divergences in modelling assumptions can translate into misleading conclusions.

People tend to have intentions concordant with healthy action. For example, people who sign up for a physical activity study will likely have stronger activity engagement intentions than the general population. This sampling bias phenomenon oftentimes manifests as a skewed intention variable distribution, in which more scores than expected are higher than the mean (e.g., ceiling effect) [30]. Distributions of physical activity behavior frequency and duration variables are rarely symmetrical either. Oftentimes, such variables are positively or negatively skewed, sometimes to the extent that they are severely inflated (i.e., more zero scores than anticipated) [31], which can lead to skewed residual distributions when used in linear models.

Digression of physical activity intention and behavior data from linear modelling assumptions is a major concern for intention-behavior gap moderation testing. This is particularly so if a hypothesized moderator variable is correlated with intention and behavior (even to a modest degree), which can exacerbate the biases from these unmet assumptions [28]. Take, for example, studies of habit. *Habit* is the process by which a person’s behavior is influenced from a prompt to act based on well-learned associations between cues and behaviors [32, 33]. As an automatic response to contextual cues, habit is expected to generate behavior in the presence of cues more rapidly and effectively than is intention, such that people are hypothesized to be more likely to act in line with habits than intentions [6, 32, 33]. Many studies have investigated whether habit could thus help explain the intention-behavior gap, such that people with weak intentions may nonetheless act where they have a strong habit for doing so [22, 34]. Most commonly, physical activity habit strength is assessed using self-reported scales reflecting the degree to which a behavior is experienced as being automatic (e.g., ‘Physical activity is something I do automatically:’ Strongly Disagree – Strongly Agree) [35, 36].

Gardner et al.’s review found that habit moderated intention-behavior associations in eight of nine available studies, such that intention-behavior associations decreased as habit strength increased [22]. Such findings are typically used to inform recommendations for promoting physical activity. Indeed, Gardner et al. concluded that “the failure of intention to translate into action where habit is strong suggests that motivation change … will not change unhealthful habits” [22]. However, physical activity habit can be highly associated with behavior and intention [32, 37]; habit typically forms through repetition of an intended action [38], such that habitual tendencies will often concur with intentions. In this case, there may be few people for whom intention is weak, yet habit is strong [34]. Therefore, the interpretation of these intention-behavior moderation findings may be misleading.

The risk for misinterpretation of significant moderation effects on intention-behavior associations is not exclusive to habit, however. It applies to the common scenario in which 1 – intention and behavior have a non-linear relationship, and 2 – the moderator variable shares substantial variability with the behavior and/or intention variable. These circumstances apply to many investigated moderator effects in the study of physical activity [34, 39] and many other health behaviors [13, 14]. Perceived behavioral control, which reflects a person’s assessment of the ease or difficulty in performing the behavior [7], provides another well-established intention-behavior moderation example. In its original conception, the theory of planned behavior postulated that perceived behavioral control would moderate intention-behavior associations, in that higher perceived control should lead to a better execution of intentions into behavior. The moderating effect of perceived behavioral control (or related constructs such as capacity) has been consistently replicated for physical activity [16] and other health behaviors [13, 14]. Meta-analyses show typically medium-sized associations between perceived behavioral control and a variety of health behavior outcomes (*r’s* = 0.31, 0.39) [13, 14] and between perceived behavioral control and intention (*r’s* = 0.54, 0.60) [13, 14]. If intention-behavior associations are asymmetrical, this questions interpretations of the observation that perceived behavioral control statistically moderates intention and behavior associations. The robustness of tests of intention-behavior moderation warrant empirical investigation.

Simulated data can shed light on issues raised when methods and their tethered theoretical implications are questioned. Simulated studies apply algorithm-generated data distributions based on pseudo-random sampling from known probability distributions of extant evidence of the variables under study; in this way, data are hypothetical, yet informed by previous empirical evidence [40]. For example, Simmons et al. used simulated data to estimate that the probability of false-positive findings in psychology experiments is likely much higher than .05, based on evidence of the breadth of flexibility of researchers’ choices about study variables and analyses [41]. This, in part, led to re-evaluation not only of specific studies, but also of popular methodology within psychology, and the promotion of conceptual replications, use of Bayesian statistics, and pre-registration of study procedures as corrective measures to improve evidence quality and validity.

## The present study

Our aim was to investigate how the interrelatedness and typical asymmetrical distributions of intention and behavior data may affect interpretations of moderators of intention-behavior associations, particularly moderators commonly aligned with intention and physical activity behavior, such as habit and perceived behavioral control. We used simulated data to compare three scenarios of physical activity intention-behavior associations and habit moderation. The comparison tested whether the common interrelatedness of these variables leads to misleading moderation conclusions. Then, we used simulated data of the same scenario with perceived behavioral control as a moderator of intention-behavior associations to demonstrate the generalizability of the findings across other moderator variables.

## Methods

Three sets of physical activity intention, behavior, and habit data and one set of intention, behavior, and perceived behavioral control data were simulated using the *MASS* function in *R* version 3.4.1 [42, 43] based on a priori set parameters with *N* = 100 with bootstrapped estimates based on 7500 replications of the raw data [44, 45]. The distribution and covariance parameters were determined based on effect sizes of meta-analyses of prospective associations [13, 14, 22, 34, 37]. All variables were set as continuous interval scales with standardized ranges. Histograms of the sampled means were visually inspected and no abnormalities were detected. All distributions were near Gaussian with very little skew (< .40). The first set – *normal, unrelated* – represented intention, behavior, and habit data with no shared variability, all normally distributed; this represents a perfect statistical model, in that it does not violate model assumptions by any degree. The second set – *normal, correlated* – met the homoscedasticity and linearity assumptions of linear modelling (i.e., normally distributed with linear relationship), but with correlated intention, behavior, and habit data to the magnitude found in previous meta-analyses. The third dataset – *realistic* – incorporated the same magnitude of correlations between intention, behavior, and habit as in the correlated set, but these were set to more closely mirror the non-normal distributions and, therefore, asymmetrical associations typical of intention-behavior relationships observed in previous physical activity research. For the test of generalizability, the intention and behavior variables were set to the same univariate parameters as in the *realistic* dataset (i.e., non-normal distributions) and the perceived behavioral control variable was set to be normally distributed, but the parameters of the correlations of perceived behavioral control with intention and physical activity behavior were set based on meta-analytic findings [13, 14]. The study was exempt from needing ethical clearance, as the data were computer-generated.

### Univariate distribution parameters

The *normal, unrelated* and *normal, correlated* simulation data distributions were set to be near Gaussian. For all models, the moderator variables of habit and perceived behavioral control were also set to be near Gaussian. For the *realistic* model and test of generalizability, the intention and behavior variable distributions were weighted based on the quartile proportions of intention-behavior profiles as found in Rhodes and de Bruijn [34]. The intentions variable was negatively skewed, reflective of most people having high scores (skewness = − 0.70, kurtosis = 3.18) and the behavior score was flattened out, reflective of fewer people having mid-range scores than would be expected with normal distribution (skewness = 0.11, kurtosis = 1.67).

### Multivariate covariation parameters

The *normal, unrelated* intention, behavior, and habit variables were all set to have null correlations (*r* ~ 0.00). For the *normal, correlated* and *realistic* sets of simulations, the intention-behavior bivariate correlation was set at *r* = 0.49, based on the meta-analytic findings of prospective intention-behavior correlational studies of physical activity [13, 46]. Across both the *normal, correlated* and *realistic* datasets, habit was set to positively associate with both behavior (*r* = .41) and intention (*r* = .49). These association effect sizes were set a priori based on systematic review and meta-analytic findings of bivariate, direct associations [16, 22, 37]. For the test of generalizability, perceived behavioral control and intention were set to correlate at *r* = .47, and perceived behavioral control and behavior at *r* = .33, in line with meta-analytic findings [13, 14].

### Moderators of the intention-behavior relationship

To test the moderating effect of habit and perceived behavioral control on intention-behavior associations, we estimated simple linear regression models with behavior predicted by mean-centered intention and moderator variables, as well as their interaction terms. The bootstrapped estimates are presented as well as the *SD* of the bootstrap estimates and the interquartile ranges (25, 75%) of the replication estimates. For illustrative purposes, moderation effects were plotted with trend lines shown for people with high (> *M* + 1 *SD*), average (< *M* + 1 *SD* & > *M* – 1 *SD*), and low (< *M* – 1 *SD*) moderator scores.

## Results

### Intention-behavior association

#### Normal, unrelated data

**Fig. 1:**
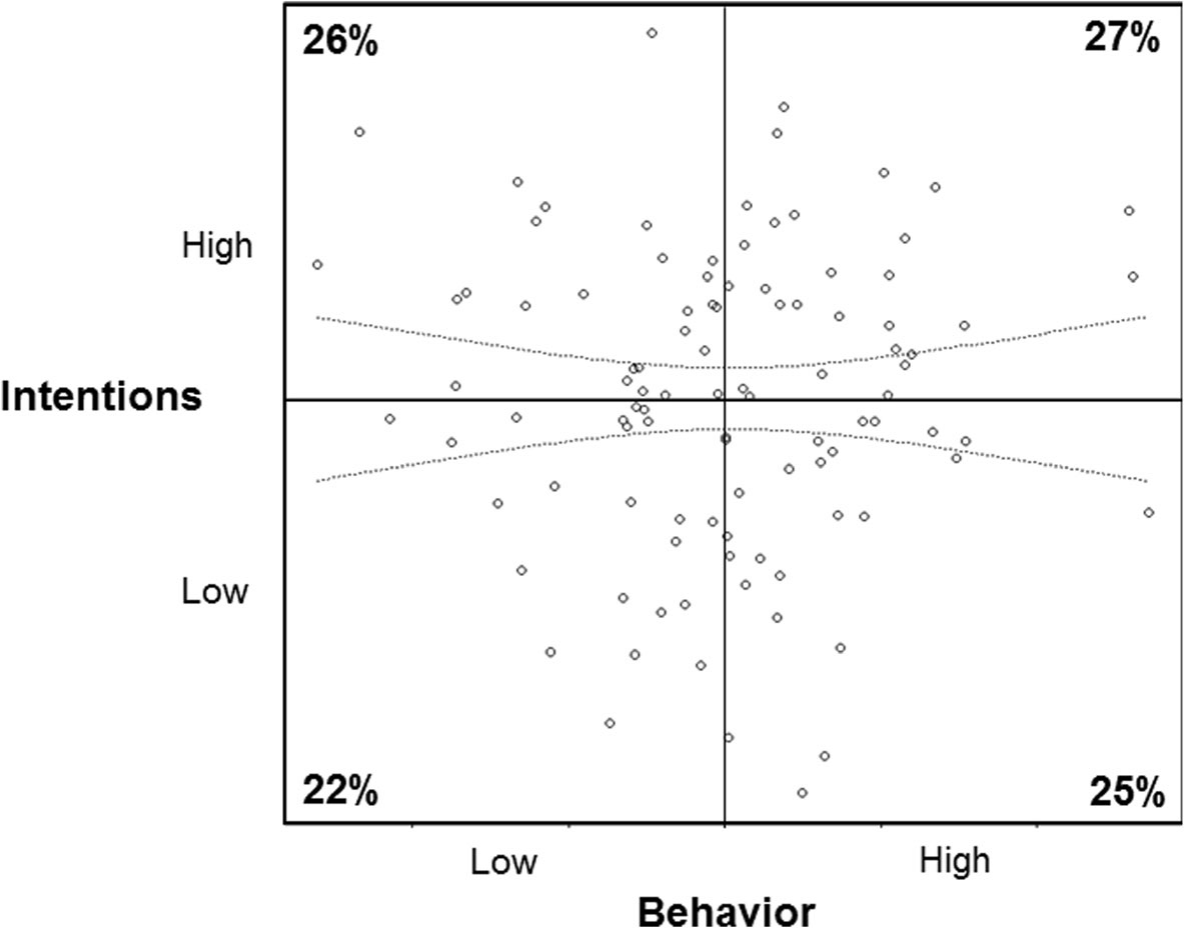
Simulated normally distributed, unrelated intention and behavior data set to near Gaussian distributions with a correlation of *r* ~ .00

Figure 1 shows the scatterplot and correlation line with +/− 2 standard error intervals of *normal, unrelated* intention-behavior data. Based on these simulations, 26% of people had high intention but low behavior scores, 27% of people had high intention and high behavior scores, 22% had low intention and low behavior scores, and 25% had high intention and behavior scores. This near-equal distribution is what would be expected with a near perfectly normal distribution, resulting in a near null association of *r* ~ 0.00 (bootstrap *SD* = 0.09).

#### Normal, correlated data

**Fig. 2:**
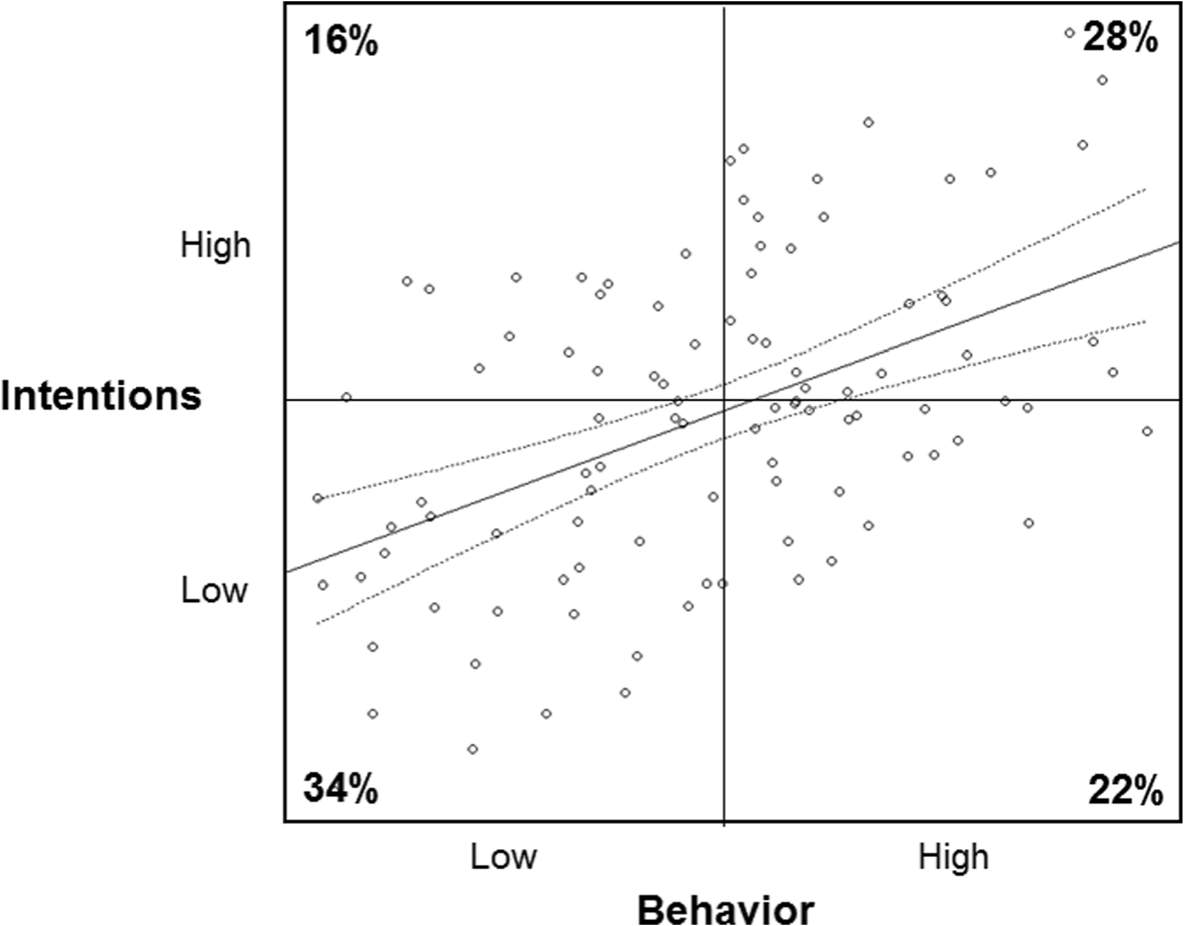
Simulated normally distributed, correlated intention and behavior data set to near Gaussian distributions with a correlation of *r* ~ .49

Figure 2 shows the scatterplot and correlation line with +/− 2 standard error intervals of the simulated intention-behavior data with normal distributions and a correlation of *r* = .49 (bootstrap *SD* = 0.09). Based on these simulations, there were only 16% of cases with high intention but low behavior scores. There were 28% of cases with high intention and behavior scores, 34% with low intention and low behavior scores, and 22% with low intention but high behavior scores. Within this fictitious scenario in which data are normal and linearly related, the intention-behavior gap is a result of both the people who made intentions and did not follow through with them (16%) as well as the 22% of people who engaged in the behavior without intention.

#### Realistic data

**Fig. 3:**
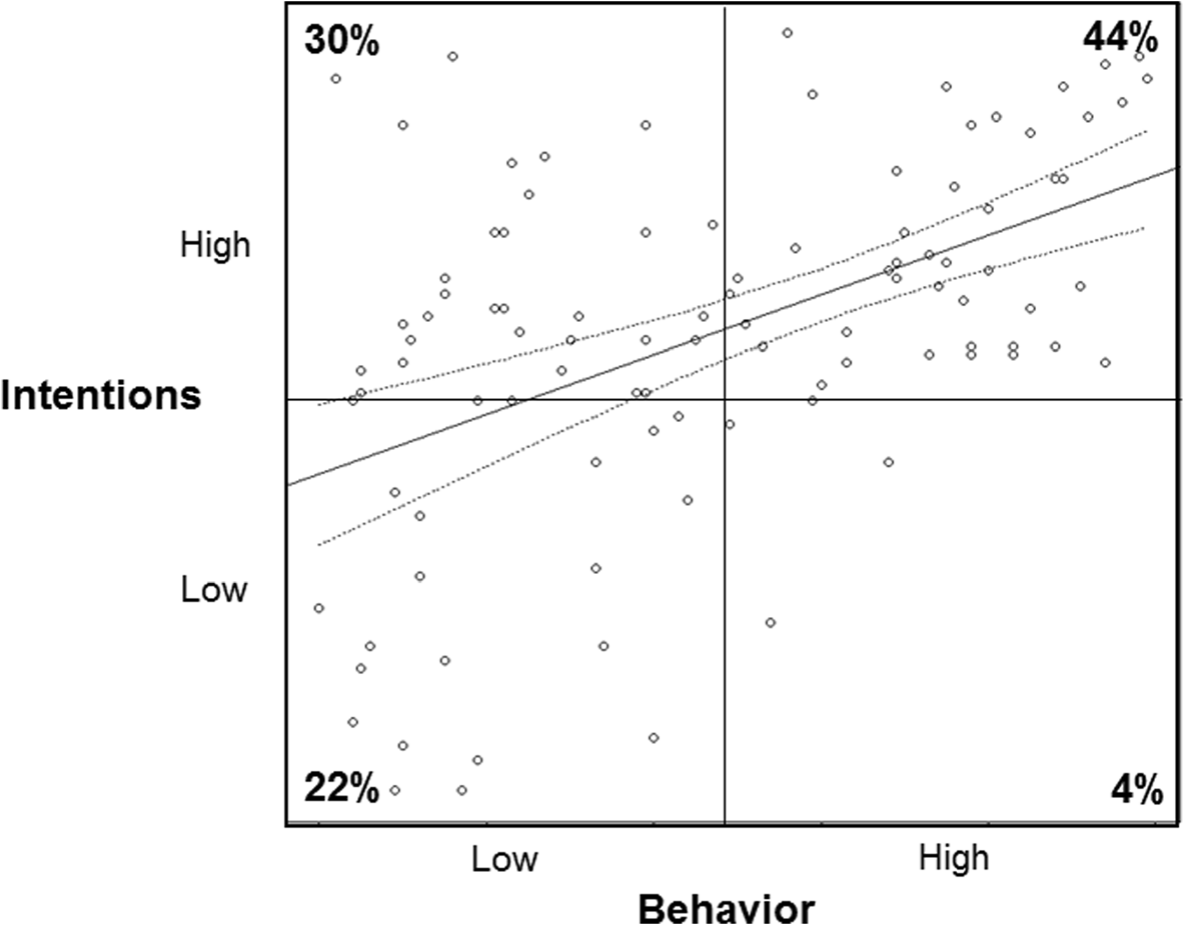
Simulated realistic intention and behavior data with non-normal distributions commonly seen in health behavior research (intention negatively skewed, behavior positively skewed) and a correlation of *r* ~ .49

Figure 3 shows the scatterplot and correlation line with +/− 2 standard error intervals of simulated intention and behavior data with the same correlation of *r* = .49 (bootstrap *SD* = .07); however the distributions were corrected to reflect the reality of typical physical activity intention and behavior data distributions as found in Rhodes and de Bruijn [34]. Compared to the simulated data with normal distributions, this scenario shows far more cases with high intention and low behavior scores (30%) and far fewer cases with low intention but high behavior scores (4%).

### Habit as moderator of intention-behavior associations

#### Normal, unrelated data

**Fig. 4:**
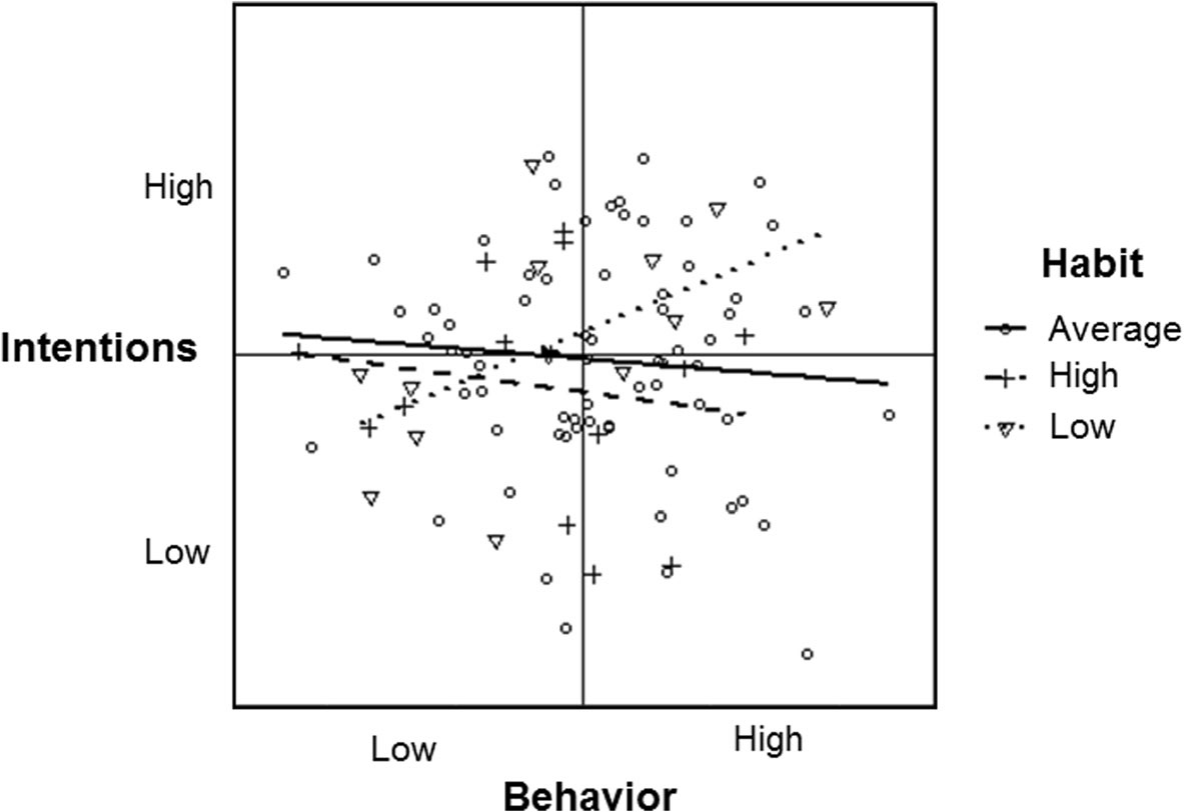
Habit moderation of the simulated normally distributed and unrelated intention and behavior data (all variables near Gaussian; *r*’s ~ 0.0)

Table 1 and Fig. 4 show a moderation effect of habit on the intention-behavior association amongst the normally distributed, unrelated dataset (*b* = − 0.17). Of the 7500 simulations, 50% of moderation effects fell within the range of *b* = − 0.24 and − 0.12. For people with low habit scores, the association between intention and behavior was *b* = 0.72 (dotted line); whereas the association was near null for people with either average (*b* = − 0.05, solid line) or high (*b* = − 0.09, dashed line) habit scores. In this instance, effects would be interpreted as evidence that the intention-behavior correlation is stronger for people with weaker habits (i.e., smaller intention-behavior gaps) than people with average or stronger habits.

**Table 1:** The Estimated Moderation Effects and Probing Analyses of Habit on the Intention-Behavior Association Applying 7500 Bootstrap Replications for Normally Distributed, Unrelated; Normally Distributed Correlated; and Realistic Intention-Behavior Data

Data	Moderation effect of Habit, *b*	Intention-Behavior, *b*	Bootstrap *SD*	Interquartile Range of Bootstrap Estimates
Normal, Unrelated	−0.17	–	0.10	− 0.24 to − 0.12
Low Habit	–	0.72	0.34	0.55 to 0.96
Average Habit	–	−0.05	0.13	−0.14 to 0.03
High Habit	–	−0.09	0.16	−0.20 to 0.01
Normal, Correlated	0.01	–	0.07	−0.05 to 0.04
Low Habit	–	0.51	0.24	0.36 to 0.65
Average Habit	–	0.44	0.10	0.37 to 0.50
High Habit	–	0.30	0.25	0.13 to 0.41
Realistic	0.07	–	0.89	−0.46 to 0.69
Low Habit	–	1.14	0.65	0.70 to 1.64
Average Habit	–	0.57	0.16	0.47 to 0.68
High Habit	–	0.58	0.15	0.48 to 0.67

*Notes. Normal, unrelated:* All variables set near Gaussian; intention-behavior *r* ~ 0.00; habit-behavior *r* ~ 0.00; habit-intention *r* = .47. *Normal, correlated:* All variables set near Gaussian; intention-behavior *r* = 0.49; habit-behavior *r* = 0.41; habit-intention *r* = .47. *Realistic:* intentions: skewness = −0.70, kurtosis = 3.18; behavior: skewness = 0.11, kurtosis = 1.67; habit and perceived behavioral control set near Gaussian; intention-behavior *r* = 0.49; habit-behavior *r* = 0.41; habit-intention *r* = .47

#### Normal, correlated data

**Fig. 5:**
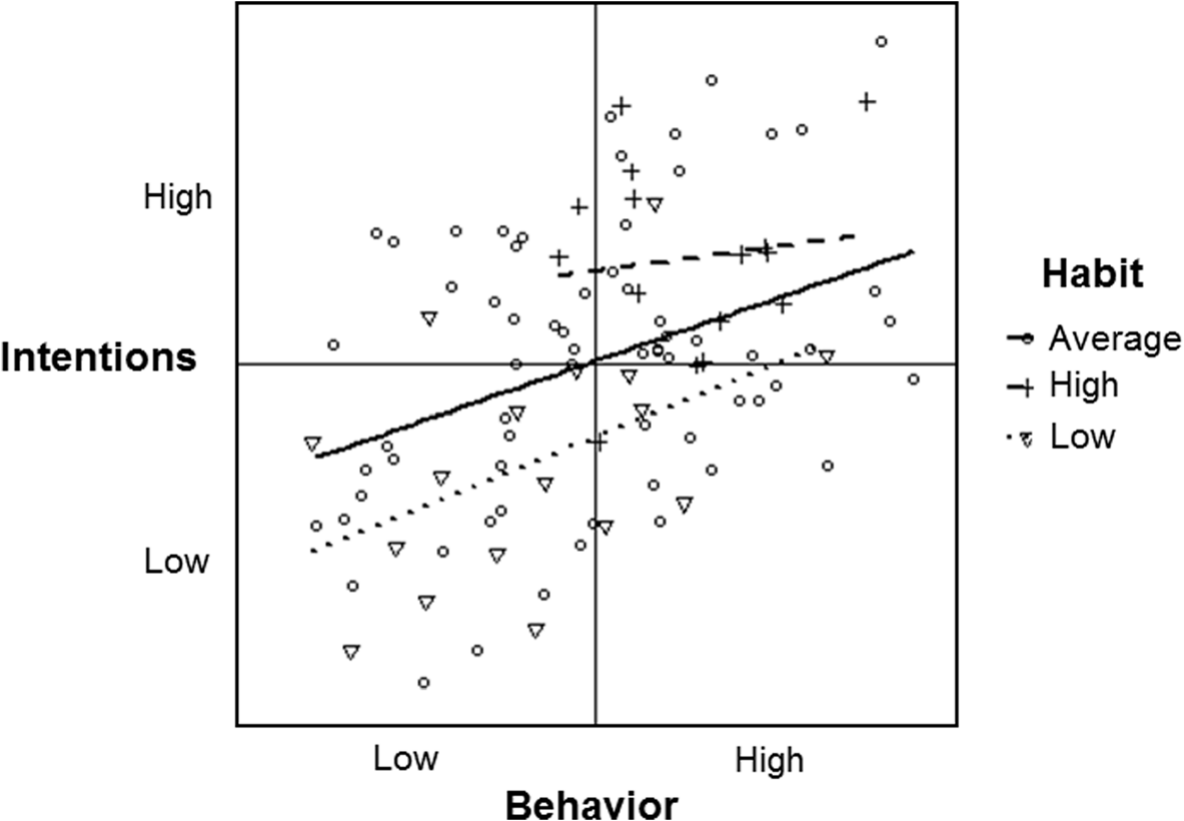
Habit moderation of the simulated normally distributed and correlated intention and behavior data (all variables near Gaussian; intention-behavior: *r* = 0.49; intention-habit: *r* = 0.49, intention-behavior: *r* = 0.41)

Table 1 and Fig. 5 show the moderation effect of habit on the intention-behavior association amongst the normally distributed, correlated dataset (*b* = 0.01). Of the 7500 simulations, 50% of moderation effects fell within the range of *b* = − 0.05 and 0.04. Under these circumstances, people with low habit scores showed an intention-behavior association of *b* = .51 (dotted line), just slightly more steep than that of the association between intention and behavior for people with average (*b* = .44; solid line) or high (*b* = .30; dashed line) habit scores. For this example, the apparent moderation effect is such that there is a slightly stronger association between intention and behavior for people with low habit scores. Of note, because the intention-behavior variables are correlated, the data range is mostly distributed in the top-right and bottom-left quadrants, such that the trend lines for habit moderation are restricted.

#### Realistic data

**Fig. 6:**
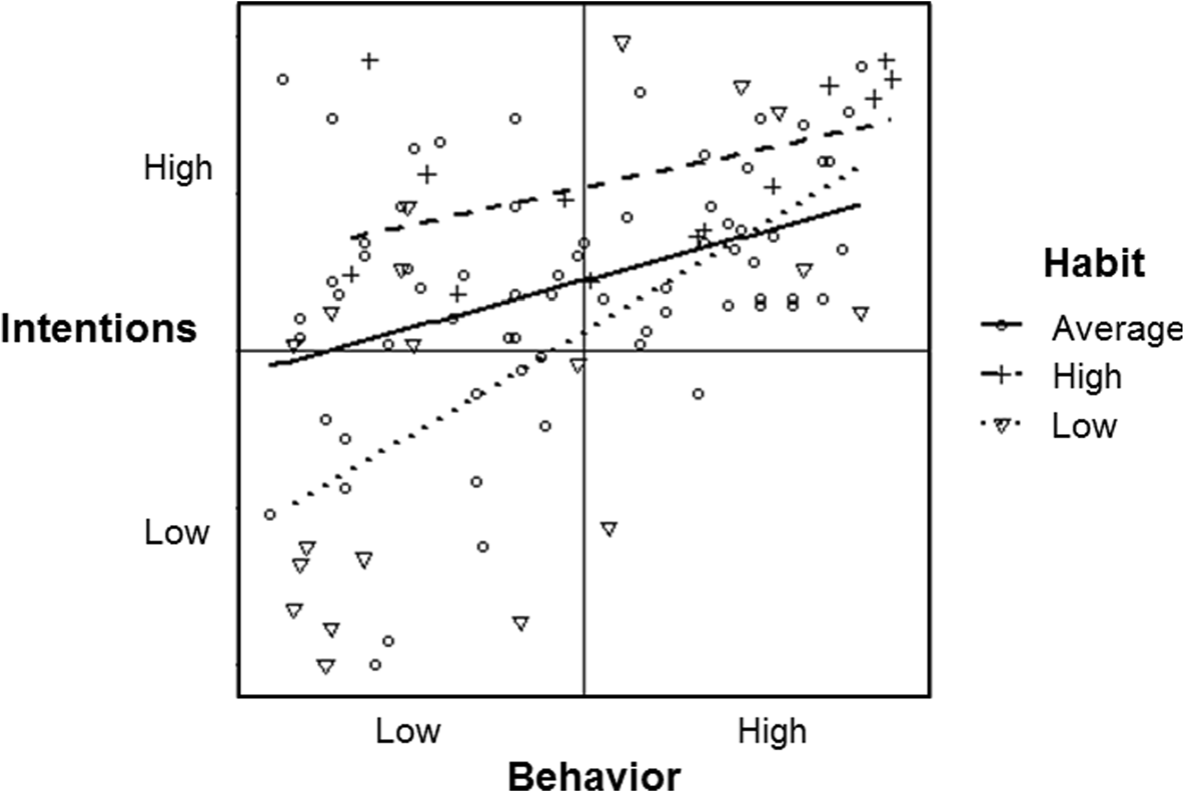
Habit moderation of the intention and behavior data simulated based on realistic distributions and correlations (intention negatively skewed, behavior positively skewed; intention-behavior: *r* = 0.49; intention-habit: *r* = 0.49, habit-behavior: *r* = 0.41)

**.** Table 1 and Fig. 6 show the moderation effect of the simulated habit variable on the intention-behavior data with realistic correlations and distributions (*b* = 0.07). Of the 7500 simulations, 50% of moderation effects fell within the range of *b* = − 0.46 and 0.69. In this instance, for cases with low habit scores, the association between intention and behavior is *r* = 0.59 (dotted line); whereas it is *r* = 0.50 (solid line) and *r* = 0.40 (dashed line) for cases with average and high habit scores, respectively. Of note, there are no cases of high habit in the bottom two quadrants (which represents people with low intention scores), so the intention-behavior association for these cases is restricted into the upper two quadrants. In contrast, the cases with low habit are also distributed on one side of the bottom half of the plot (which represents people with low intention and behavior scores), allowing for a less restricted range of association.

### Perceived behavioral control as moderator of intention-behavior associations

**Fig. 7:**
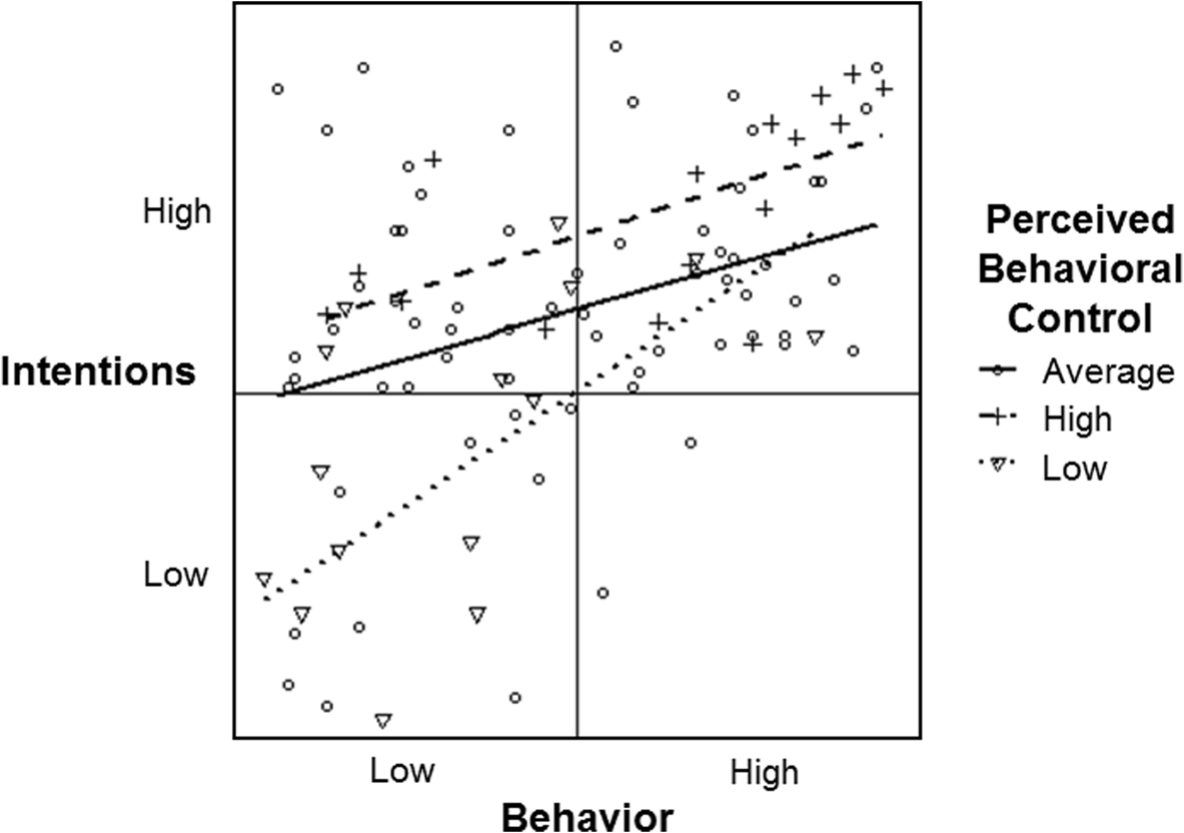
Perceived behavioral control moderation of the intention and behavior data simulated based on realistic distributions and correlations (intention negatively skewed, behavior positively skewed; intention-behavior: *r* = 0.49; intention-perceived control: *r* = 0.47, perceived control-behavior: *r* = 0.33)

Figure 7 shows the moderation effect of the simulated perceived behavioral control variable on the intention-behavior data (*b* = 0.70). Of the 7500 simulations, 50% of moderation effects fell within the range of *b* = 0.26 and 1.77. For cases with high perceived control scores, the association between intention and behavior is *r* = 0.63 (dashed line), which is nearly twice as strong as it is for those with average perceived control scores (*r* = 0.34; solid line). However, as a result of the asymmetry of the intention-behavior data, those with low perceived behavioral control scores also have a strong intention-behavior association at *r* = 0.58 (dotted line). This effect demonstrates that the risk for overextrapolation of intention-behavior moderation effects to those seldomly represented within the data (i.e., those with high behavior but low perceived behavioral control scores) generalizes beyond habit to other moderator variables, as a result of asymmetrical intention-behavior associations.

## Discussion

The asymmetry of intention-behavior relationships is well-documented in physical activity research; far more people fail to act on strong intentions than are frequently physically active despite weak intentions [15, 19]. Our study provides an innovative demonstration of how this asymmetry can lead to potential misinterpretation of intention-behavior associations and investigations of moderators of these associations. We used simulated data based on normal distributions with no shared variance, correlated parameters with normal distribution, and realistically correlated and non-normally distributed parameters. Comparing the three scenarios, we illustrated how the same correlation coefficient may mask different types of intention-behavior associations. Specifically, we showed that the typical patterns of intention and behavior data in physical activity research leads to violations of two fundamental assumptions of linear modelling. These findings highlight a risk of misinterpreting testing of moderation of the intention-behavior gap for physical activity.

The prevalent risk of misinterpretation of intention-behavior associations (and moderation thereof) may impact on the implementation of physical activity behavior change interventions. The public health benefits of regular physical activity are numerous and most interventions focus on intention-enhancing strategies such as education, risk awareness, goal-setting and self-monitoring [2]. If the science underpinning our understanding of how intentions translate into behavior is misinformed, then we may be targeting the wrong behavior change strategies (e.g., intention formation instead of action control) or tailoring our efforts based on a non-existent population sub-group (e.g., those with low intention but high behavioral engagement).

The standard assessment of moderation of the intention-behavior gap is reliant on correlation coefficients of linear regression, which do not capture the intention-behavior profiles that underpin the intention-behavior gap. If effects were applicable to the entire available data range, the gap would be made of a near equal ratio of people who were intending to engage in the behavior and subsequently did not, and those with no intention who nonetheless engaged in the behavior. Yet this is not usually the case, at least for typical physical activity intention-behavior studies; the stability of the intention-behavior correlation is mostly the result of non-intenders doing nothing. Even if people intended to act, there is still nearly the same probability of the behavior being enacted as winning a coin toss (46%) [34]. This is interesting because, while it partly supports theories of intention [7, 9–11], it is not helpful for promoting physical activity. This additional information about the reality of intention-behavior associations supports action control theories [19, 47], by suggesting intention is necessary but not always sufficient for physical activity.

After illustrating how typical physical activity intention-behavior data deviate from linear associations with normally distributed residuals, we demonstrated how this asymmetrical association restricts the range of data available for estimating the extent to which a moderator may impact intention-behavior relationships, particularly if the moderator is associated with intention or behavior [28]. Under these circumstances, significant moderation correlation coefficients should not be interpreted as generalizable to people who engage in physical activity without intention, because those people seldom exist.

The commonly tested moderation of habit on the intention-behavior gap in physical activity research is an example of such a circumstance. Theory predicts that habit will override the effect of intention on behavior [6], an effect that has been found in many previous studies [22, 37]. From a theoretical perspective, this should reflect that people with strong habits unintentionally engage in physical activity, because regulation is shifted from the conscious processes needed for intentional action to the automatic cue-to-action processes that elicit habitual responses [48]. This assumption has generated much interest in habit formation interventions as a potential means to behavior maintenance [49, 50]. Commentators have reasoned that, because habit overrides intention, people should be encouraged to make any new physical activity regimens habitual, because they will likely sustain such activity in the face of typical losses in motivation over time [51]. Yet, the evidence base on which this is assumption is based, which appears to show that the effect of intention on behavior weakens as habit strength increases, may be based on statistical byproducts of unmet model assumptions [52]. In such studies, there are typically few people with strong habit but weak intention and almost nobody appears to engage in unintended physical activity behavior. This refutes the idea that those with habits act *without* intention. This issue was pointed out by Rhodes et al. in their tests of the habit, intention, and physical activity relationship using linear regression compared to a comparison of people categorized based on whether they achieved their intention or not (i.e. which quadrant of the scatterplot their data point fell within) [52]. What they found, and subsequently replicated several times [34], is that, among people with stronger intentions, habit helps to translate intentions into action (presumably by minimizing demands on memory and other attentional resources) [6]. This is perhaps unsurprising: realistically, habit likely forms on the basis of consistent repetition of intentional action [38], such that habits and intentions often correspond [53]. A more nuanced perspective on the practical value of habit formation for behavior maintenance emerges: habit may assist in driving physical activity when people with strong intentions experience dips in motivation (e.g., a habitual distance runner in bad weather conditions). Habit is, however, unlikely to facilitate engagement in activity among people with no intention to be active, or those who strongly intend not to be active. This may explain why, contrary to theory, some research into longer-term effects of habit-formation interventions has observed declines in the focal behavior over time, despite apparent gains in habit strength [54].

Our data should not, however, be taken to indicate that habit never overrides intentional tendencies. Although our data were simulated to reflect the typical concurrence of habit and intentions in the physical activity domain, there are nonetheless valid real-world instances in which habits conflict with intentions. For example, people often form intentions to tackle their bad eating or smoking habits [55–57]. Such instances of discordance between intentions and habits may offer more credible settings for estimating the moderating impact of habit on intention-behavior relationships. Although more studies of counter-habitual intentions are needed, it is however notable that the few such studies to date have found little evidence to suggest that habit moderates the intention-behavior gap [35, 55, 56, 58].

We also demonstrated that the same misinterpretation risk generalizes to variables other than habit by showing similar findings using perceived behavioral control. Whereas theory postulates that higher perceived control translates into more effective action control [7], our findings show how these significant moderation effects may be statistical byproducts of asymmetrical intention-behavior relationships. Other moderators of the intention-behavior gap in physical activity likely also will be impacted in the same manner. For example, *implementation intentions* – specific actionable plans about what, where, how, and when intentions will be implemented – are oftentimes proposed as a mechanism by which intentions translate into behavior [59–61]. Given that amongst these studies, implementation intentions and intentions are oftentimes strongly associated, such moderation analysis is susceptible also to misleading conclusions from these unmet assumptions of linear modelling.

### Recommendations for future intention-behavior research

We have some recommendations for how future intention-behavior research could check whether their intention-behavior data are asymmetrical and for managing the imposed risk for misinterpretation of the intention-behavior gap and tests of moderation. These limitations presented here only apply to asymmetrical intention-behavior relationships resulting from non-normal distributions so will not be applicable across all scenarios.

During study development, simple adjustments to recruitment strategy and measurement may make these violations of linearity assumptions less likely. Common physical activity study recruitment methods are prone to oversampling those with more positive activity intentions (e.g., flyers placed in gyms, or recruitment of students enrolled on movement-based university courses). Researchers should engage in recruitment efforts less targeted toward those with strong activity intentions, to increase the likelihood of more normally distributed intention data. Additionally, ensuring an intention measure provides a reasonably broad range of possible response options may reduce the risk of ceiling effect [62]. For example, our experiences of piloting intention measures show that, instead of asking whether people will engage in a set amount of physical activity, more normally-distributed intention data can result from using an open response item requiring respondents to report how much time they intend to be active in a set period. Consideration for the measurement of potential confounders and moderators of intention-behavior associations is also important. For example, some self-report measures of habit strength include items assessing behavioral frequency, which will result in shared variability between habit and behavior variables [36], therefore exacerbating the risk of asymmetrical relationships. If the aim is to investigate the moderating role of habit in intention-behavior associations, alternative measures which do not include the behavioral frequency items may be more applicable [35, 63].

Following data collection, efforts can also be made to reduce risks of misleading findings. Assumption testing is essential to ensure the estimates from analyses are interpreted correctly [27–29]. The most prudent assumption testing method harkens back to introductory statistics courses: plot the data. By visualizing the correlation, one can assess whether either intention or behavior data deviates from the normal distribution curve as well as whether there is asymmetry in how the data are dispersed across each of the four quadrants. Unfortunately, past evidence shows that the pattern of asymmetry simulated here is common for intention-behavior associations amidst physical activity research [34]. Fortunately, there are simple ways to address the commonly unmet assumptions of homoscedasticity and linearity.

If the intention-behavior association does seem asymmetrical, it may be tempting to just transform the non-normal variable (e.g., if physical activity is positively skewed, many people square root or log transform it). Although this may adjust for the statistical modelling violation, it leads to uncertainty when it comes to making meaningful conclusions from the finding. Univariate transformations do not account for the practical problem that we may be generalizing findings to non-existent people (e.g., people who engage in physical activity without reporting intention). There are many more appropriate techniques for managing these assumption violations; which technique is most applicable depends on the research aims, design, and measurement factors.

When the study aim is to investigate decisional intentions (and the measurement reflects that), an option for testing intention-behavior associations and moderators may include profile analyses [15, 19, 64]. This method involves categorizing cases based on the 2 × 2 matrix (analogous to the four quadrants in the scatterplots) based on 1 – the decisional intention to engage in the behavior or not, and 2 – whether they subsequently engaged in the intended amount of the behavior or not. The matrix with these category labels from Rhodes and de Bruijn [19] and Sheeran [15] are presented in Table 2. Upon categorizing people into these profiles, the nominal group variable (of which there are four categories) can then be entered as a predictor or outcome in any applicable model. For example, one may test whether habit predicts peoples’ intention-behavior profiles. This option may be particularly relevant for health behaviors for which there are evidence-based guidelines around which a dichotomy might be constructed, such as physical activity (i.e. whether 150 min per week of moderate-to-vigorous activity is achieved).

**Table 2:** The 2 × 2 Matrix of Profile Analysis Based on Decisional Intentions and Subsequent Behavior

	Did they engage in the intended amount of behavior?	
Did they intend to engage in the set amount of behavior?	No	Yes
No	• Non-intenders • Disinclined abstainers	• Non-intenders exceeding intentions • Disinclined actors
Yes	• Unsuccessful intenders • Inclined abstainers	• Successful intenders • Inclined actors

Notably, it can be difficult to identify appropriate cut-off values that are required by profile analyses. Additionally, by categorizing continuous measurements, profile analyses can limit precision and sensitivity [65, 66]. Given its limitations, we do not recommend the replacement of intention-behavior moderator testing using continuous variables with profile analysis; rather, we recommend using profile analysis to test the generalizability of conclusions drawn from linear moderation estimation. Concordant findings across both analyses would provide more certainty of the meaningfulness and generalizability of the findings. However, if the findings were discrepant, it may indicate the linear moderation is only representative of a certain sub-group of the sample, which could be ascertained via profile analysis.

If supplemental profile analysis is not well-suited for the research question, nonlinear regression may be more suitable for testing intention-behavior associations and potential moderators of the association. This can be applied with the *nlstools* in *R* [67, 68]. The obvious benefit of non-linear modelling is that it is not reliant on the assumptions underpinning linear modelling, although it is worth noting that nonlinear regression is dependent on its own set of assumptions being met, so the appropriate diagnostics are necessary beforehand. Additionally, it should be noted that sometimes physical activity behavior (and even intention) data take the form of frequency counts (e.g., number of bouts per week/day), in which case the most appropriate analysis strategy would be to account for the Poisson distribution(s) with Poisson (for overdispersion) or negative binomial (for underdispersion) analyses [69]. Given the asymmetry common of health behavior intention-behavior relationships, future research may consider establishing the trajectory of intention-behavior associations and modelling it appropriately (e.g., by incorporating exponential term in regression).

## Conclusions

Advancements in our understanding about what motivates people to engage in physical activity is essential in the global effort to reduce the costly burden of global inactivity [2]. Understanding psychological determinants of physical activity behavior requires understanding not only people’s intentions, but also the factors that affect the likelihood of them acting on their intent. The emerging evidence on the intention-behavior gap and its moderating factors is promising, but this study illustrated how the typical patterns of intention and behavior data in physical activity research leads to violations of fundamental assumptions of linear modelling. As a result of these violated statistical assumptions, there is a risk that we are misinterpreting findings important for developing effective physical activity promotion efforts. For example, we have demonstrated that the hypothesis that the effect of intention on behavior weakens as habit strength increases may be based on statistical byproducts of unmet model assumptions. The generalizability of these findings was supported through the replication of the findings with the test of the moderating effect of perceived behavioral control on intention-behavior relationships. To ensure research is practically relevant at a behavioral medicine level, research of intention-behavior associations and moderation of the intention-behavior gap need to be considerate of the risk for misinterpretation from the asymmetry of the real-world phenomenon of intention-behavior associations.

## Data Availability

The datasets used and/or analysed during the current study are available from the corresponding author on reasonable request.
